# Éviscération vaginale post hystérectomie récidivante

**DOI:** 10.11604/pamj.2014.19.42.4366

**Published:** 2014-09-18

**Authors:** Boubacar Zan Traoré, Anis Benmansour, Hajar Hachim, Oumar Saoud, Jean Claude Ekogha Ekogha, Rachida Mbida, Rachid Mohammed Chkoff

**Affiliations:** 1 Clinique Chirurgicale des Urgences Viscérales Hôpital Avicenne CHU Rabat- Salé, Rabat, Maroc

**Keywords:** Éviscération vaginale, Récidive, Urgence chirurgicale, vaginal evisceration, recurrence, surgical emergency

## Abstract

L’éviscération vaginale après hystérectomie, bien que rare, est une complication postopératoire grave pour les patients jeunes et les personnes âgées. Chez la femme ménopausée, elle est très souvent due à une chirurgie pelvienne, associée à un trouble de soutien du plancher pelvien. L'iléon distal est l'organe le plus fréquent d’éviscération, mais le prolapsus de l’épiploon, appendice et des trompes de Fallope a été rapportés. Une patiente âgée de 53 ans était admise au service des urgences chirurgicales viscérales en raison d'une masse vaginale apparue sans effort notable. Ses antécédents comportaient une hystérectomie vaginale réalisée en 2009 pour une pathologie bénigne et une éviscération vaginale en 2011 traitée par voie vaginale. L'examen gynécologique révélait une éviscération vaginale d'anses intestinales par une rupture du sommet du vagin. Le segment intestinal hernié, sale, œdématié avec infiltration de la graisse mésentérique est jugé viable. La patiente était opérée en urgence immédiatement après le diagnostic par laparotomie médiane sous-ombilicale. La réintégration en douceur de l'intestin permettait d'objectiver une perforation du sommet du vagin. Cette dernière était fermée par un surjet au fil résorbable après ravivement des berges de la perforation. Le vagin était ensuite fixé au promontoire par des points séparés. Les suites opératoires étaient simples. La patiente sortait de l'hôpital après six jours d'hospitalisation. L’éviscération vaginale est une urgence chirurgicale dont les modalités de prise en charge doivent être connues. Si la laparotomie a longtemps été la technique de référence, la voie vaginale exclusive peut être réalisée en cas d'exploration aisée du segment éviscéré.

## Introduction

L’éviscération vaginale après hystérectomie, bien que rare, est une complication post-opératoire grave pour les patients jeunes et les personnes âgées. Chez la femme ménopausée, elle est très souvent due à une chirurgie pelvienne, associée à un trouble de soutien du plancher pelvien. L'iléon distal est l'organe le plus fréquent d’éviscération, mais le prolapsus de l’épiploon, appendice et des trompes de Fallope a été rapportés. Le but ce travail est mettre l'accent sur la difficulté d'adopter une attitude thérapeutique.

## Patient et observation

**Figure 1:**
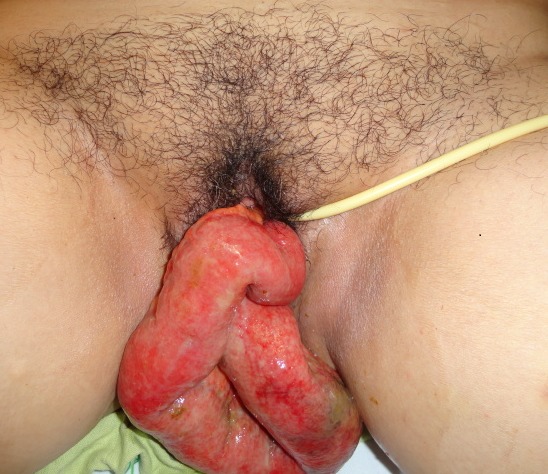
Éviscération trans- vaginale de l'intestin grêle

**Figure 2:**
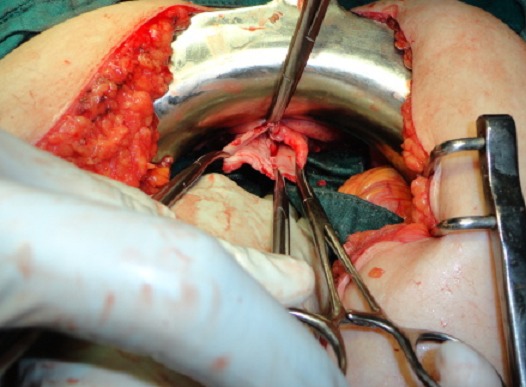
Éviscération trans-vaginale: Vue opératoire de l'ouverture vaginale trans-péritonéale

Une patiente âgée de 53 ans était admise au service des urgences chirurgicales viscérales en raison d'une masse vaginale apparue sans effort notable. Ses antécédents comportaient une hystérectomie vaginale réalisée il y à cinq ans pour une pathologie bénigne, une éviscération vaginale traitée chirurgicalement par voie vaginale il y à trois ans. L'examen gynécologique révélait une éviscération vaginale d'anses intestinales par une rupture du sommet du vagin. L'anse intestinale hernié, œdématiée avec infiltration de la graisse mésentérique est jugée viable (Figure 1) après une inspection minutieuse. La patiente était opérée en urgence immédiatement après le diagnostic par laparotomie médiane sous-ombilicale. La réintégration en douceur de l'intestin permettait d'objectiver une perforation du sommet du vagin (Figure 2). Cette dernière était fermée par un surjet au fil résorbable après ravivement des berges de la perforation. Le vagin était fixé au promontoire par des points séparés. Les suites opératoires étaient simples. La patiente sortait de l'hôpital après six jours d'hospitalisation.

## Discussion

L’éviscération vaginale après hystérectomie est une complication rare et peu connu en chirurgie gynécologique. Chez la femme ménopausée le vagin est mince, cicatriciel, avec une vascularisation précaire qui le rend plus exposé pour la rupture [1]. La rupture se fait le plus souvent à la partie postérieure du fornix [2, 3]. Après la ménopause, l’éviscération transvaginale peut être spontanée comme dans notre observation, ou secondaire à une augmentation brutale de la pression intra-abdominale (toux, défécation, chute). Les interventions vaginales et l'existence d'un entérocèle [4, 5], sont des facteurs de risque. Dans la série de Kowalsky et al. à propos de 60 cas, 73% des malades avaient des antécédents de chirurgie vaginale, la plus généralement commune était l'hystérectomie transvaginale ou une cure d'entérocèle par voie transvaginale. La rupture spontanée serait due à l’étirement progressif d'un vagin atrophique [4, 5, 6].

L′éviscération vaginale est une urgence chirurgicale et sa prise en charge consiste à repositionner les anses intestinales en intra abdominal et une inspection minutieuse pour vérifier l′intégrité des anses intestinales. Dans de rares cas, une résection intestinale est nécessaire. La procédure nécessite généralement une laparotomie, comme dans notre expérience, même si certains préconisent une approche vaginale simple pour les premiers cas [7]. Narducci et al. [8] rapporte deux cas réparés avec une laparoscopie combinée et voie vaginale utilisant un épiplooplasties sans complication post-opératoire.

## Conclusion

L’éviscération vaginale est une urgence chirurgicale dont les modalités de prise en charge doivent être connues. L'incidence globale est faible, mais non négligeable; par conséquent, le diagnostic et le traitement de cette complication post- hystérectomie doivent être gardés à l'esprit dans un centre ayant une grande activité chirurgicale.

## References

[1] Lina O'Brien, Lisa Bellin, Gerald Isenberg (2002). Spontaneous transvaginal small-bowel evisceration after perineal proctectomy. Dis colon.

[2] Friedel W, Kaiser IH (1975). Vaginal evisceration. Obstet Gynecol..

[3] Rolf BB (1970). Vaginal evisceration. Am J Obstet Gynecol..

[4] Kowalski LD, Seski JC, Timmins PF, Kanbour AI, Kunschner AJ, Kanbour-Shakir A (1996). Vaginal evisceration: presentation and management in postmenopausal women. J Am Coll Surg..

[5] Gordon PH, Nivatvongs S (1999). Principles and practice of surgery for the colon, rectum, and anus.

[6] Powell JL (1973). Vaginal evisceration following vaginal hysterectomy. Am J Obstet Gynecol..

[7] Cardosi RJ, Hoffman MS, Roberts WS, Spellacy WN (1999). Vaginal evis- ceration after hysterectomy in premenopausal women. Obstet Gynecol..

[8] Narducci F, Sonoda Y, Lambaudie E, Leblanc E, Querleu D (2003). Vaginal evisceration after hysterectomy: the repair by a laparoscopic and vaginal approach with a omental flap. Gynecol Oncol..

